# “A Review of *Parquetina nigrescens* (Afzel.) Bullock, A Plant for Traditional Medicine: Phytochemical and Pharmacological Properties”

**DOI:** 10.1155/2022/6076707

**Published:** 2022-11-16

**Authors:** Emmanuel Adase, Peter Ankutse, Doris Kumadoh, Mary-Ann Archer, Michael O. Kyene, Genevieve N. Yeboah, David Okata Asamoah Agyare

**Affiliations:** ^1^Department of Production, Centre for Plant Medicine Research, Mampong-Akuapem, Ghana; ^2^Plant Protection and Regulatory Services Directorate, Pokuase, Ghana; ^3^Department of Pharmaceutics, Centre for Plant Medicine Research, Mampong-Akuapem, Ghana; ^4^Department of Pharmaceutics, University of Cape Coast, Cape Coast, Ghana; ^5^Industrial and Commercial Department, Centre for Plant Medicine Research, Mampong-Akuapem, Ghana

## Abstract

The development of herbal medicines as a remedy for several illnesses has been recognized and accepted worldwide by health experts. *Parquetina nigrescens* is a perennial evergreen woody climber from the *Apocynaceae* family, widely used in Africa for the treatment of many diseases. The current study is intended to review and put together information available on this ethno-medicinal plant, which will improve scientific knowledge about the plant and also identify research areas that need to be investigated further. The information related to the plant was obtained using scientific databases such as Google scholar, WebMD, Wiley, Science direct, Cochrane database, student thesis, PubMed, and Scopus to obtain relevant literature regarding the botanical descriptions and distribution of the plant, traditional uses, phytochemicals, active compounds isolated from the plant, and pharmacological properties of *P. nigrescens.* Several traditional uses for different parts of the plant (leaves, stem bark, roots, leaf sap, flowers, and latex) have been presented. A review of the phytochemical composition of different plant parts revealed the presence of reducing sugars, flavonoids, tannins, alkaloids, cardiac glycosides, steroids, coumarin, anthraquinones, terpenoids, and saponins. Many studies also highlighted pharmacological activities related to *P. nigrescens,* including antianemic and haematological activity, antidiabetic, anti-inflammatory, antipyretic, analgesic, antiasthmatic, antimicrobial, insecticidal, neurotoxic, cytotoxic, antityphoid, antipolycystic ovarian syndrome activity, antilipidemic, and memory-enhancing activity. It is recommended that further *in-vivo* and clinical studies be conducted on the plant for the development of novel drugs.

## 1. Introduction

Herbal medicines have existed since the beginning of mankind. They play an important role in battling ill-health problems. Several civilizations have records of the pioneers of herbal medicine. The first Emperor of Ancient China, Shennong Yan, was credited with having invented herbs for treating his people's illnesses. It is also said that Shennong tried all kinds of herbs, found medicinal materials, and taught people to treat diseases [1]. Today, the evidence is so clear that the application of plants or plant materials in medicine has been recognized. In some African communities, herbal medicines are used as first aid for common conditions like malaria, fever, and colds. There is a lot of evidence on the toxicological aspect of some plants, indicating they could be detrimental to humans' health when consumed irrationally. It is important that plants are studied carefully for both efficacy and safety to assure public health and safety. The World Health Assembly highlighted the need to ensure the quality of medicinal plant products by making use of current quality control techniques and using suitable standards [2]. *Parquetina nigrescens* has been used for the treatment of several diseases in many African countries, including Ghana, Nigeria, and Senegal. [3]. Several studies have been conducted on the phytochemical constituents and pharmacological activities of the plant. The plant has been found to possess alkaloids, cardenolides, and glycosides [4]. Studies have revealed antioxidant, anti-inflammatory, antisickling, haematological, cytotoxic, antimicrobial, antipyretic, sympathomimetic, uterotonic, hematopoietic, analgesic, and antiulcerogenic properties of the plant [5, 6]. There is currently no review that puts all the data on the various reported activities of the plant together. This article intends to gather all the data regarding the botanical description and distribution, traditional uses, phytochemical constituents, active compounds or secondary metabolites, and isolated and pharmacological activities of the plant to enable ease of further research on the plant for likely disease conditions of interest. The review will also provide a possible source for the pharmaceutical industry to develop novel drugs for disease treatment, as well as improve scientific knowledge about the plant and identify research areas that need to be investigated further.

## 2. Methodology

The information for this review was obtained from available literature (online and offline) using electronic data and scientific research information resources such as Scientific Information Database, PubMed, WebMD, Wiley online library, Cochrane Database, Scopus, Springer Link, and African Journals on Line, as well as Google scholar and student thesis. In addition, educational newspaper articles were also used. Parquetina nigrescens; Botanical description and distribution of *Parquetina nigrescen;* scientific synonyms of *Parquetina nigrescens*; traditional uses of *Parquetina nigrescens*; phytochemical constituents of extracts of *Parquetina nigrescens*; isolated and active compounds from *Parquetina nigrescens;* pharmacological activities (including antianemic, antidiabetic, antihypelipidemic, antioxidant, antiulcerogenic, antisickling, antimalaria, antidiarrhoea, anti-inflammation, antipyretic and analgesic, antiasthmatic) of *Parquetina nigrescens*; antimicrobial activity of *Parquetina nigrescens;* toxicological activity of *Parquetina nigrescens* were the search terms used in the retrieval of information. The search was conducted over a seven-month period and included references published from 1818 to May, 2022. Also, both *in-vitro* and *in-vivo* pharmacological tests conducted on the various plant parts, extraction solvents, and models used were included. The documents that met the criteria of the review paper were recovered and evaluated by the authors.

## 3. Results

The search of the available literature revealed no detailed review research on the *in-vitro* and *in-vivo* activities of the plant. Some of the information on the *in-vitro* and *in-vivo* activity and the related pharmacological activities of the plant, as well as botanical description and distribution, phytochemical constituents and isolated compounds from the plant, and traditional uses, has been presented.

### 3.1. Botanical Description and Distribution of *Parquetina nigrescens*

**Figure 1:**
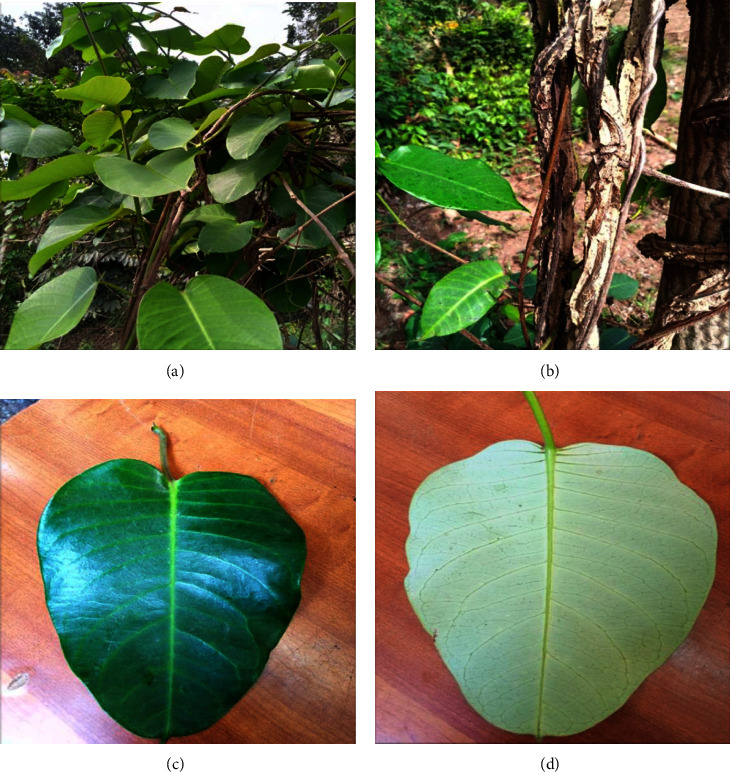
(a). *P. nigrescens* full plant (b). Climbing stem of *P. nigrescens*. (c). Adaxial (Upper surface) of leaf (d). Abaxial (Lower surface) of leaf. a–d: Source: A photograph of a full plant of *P. nigrescens*, including the climbing stem, adaxial leaf, and abaxial leaf, at the Centre for Plant Medicine Research (CPMR) arboretum in Mampong-Akuapem, Ghana.

**Figure 2:**
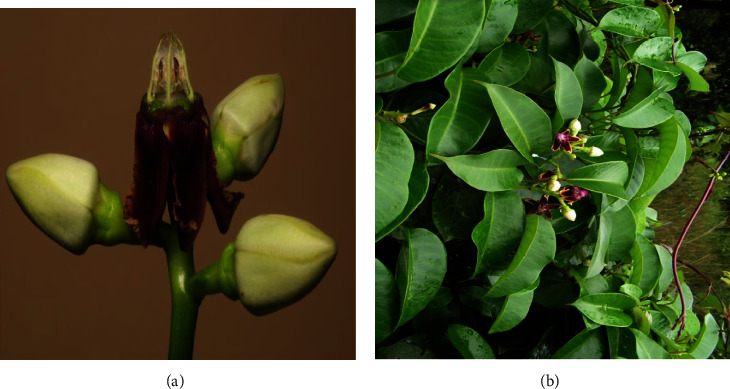
(a). *P. nigrescens* flowering. (b). *P. nigrescens* flowers on the plant. Figure 2a: Source: https://www.westafricanplants.senckenberg.de/images/pictures/parquetina_nigrescens2_cut_sl3614_1211_7a190d.jpg. Figure 2b: Source: https://www.westafricanplants.senckenberg.de/images/pictures/apocynaceae_parquetina_nigrescens_1_josstev_1211_777333.jpg.

**Figure 3:**
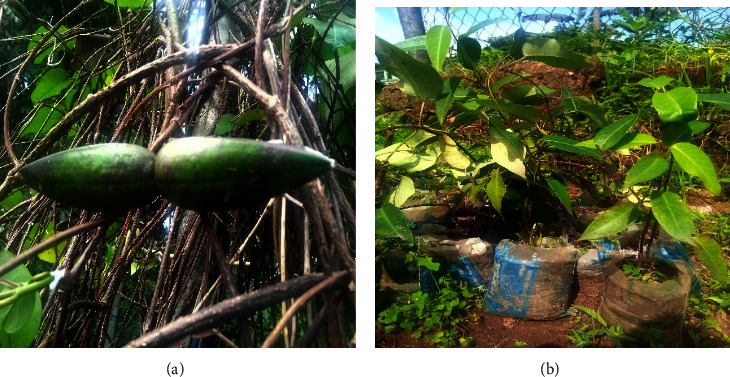
(a). *P. nigrescens* fruits. (b). *P. nigrescens* seedlings in a nursery. a-b: Source: A photograph of P. nigrescens fruits and seedlings at the Centre for Plant Medicine Research (CPMR) arboretum in Mampong-Akuapem, Ghana.

Parquetina nigrescens is a perennial evergreen woody climber plant with a twinning stem, a ligneous base of about 10–15 cm long and 6–8 cm wide, and a long smooth stem that produces the leaves (Figure 1). The leaves are glossy in texture and twine on other plants or rock surfaces, ant-hills, marshy areas, savannas, and gallery forests. The inside of the fresh coriaceous corolla normally has a maroon, pink, or deep crimson to black-violet coloration [3, 7, 8]. The leaves are mostly cordate in shape, with a length of 6.5 cm–18.3 cm and a width of 5.4 cm–16.8 cm, and pinnately veined. The leaf is normally odourless with a light green abaxial colour, a dark green adaxial colour, and an acuminate apex (Figure 1) [9]. Its flowers develop from the sides of the branches and have a whitish coloration outside and a reddish coloration inside (Figure 2). The fruit has pubescent seeds, the outer coat being woody and the inner coat softer (Figure 3). Stamens are shaggy with tetrad pollen [10]. *P. nigrescens* can be reproduced through both sexual and asexual reproduction methods using stem cuttings or seed. The germinated seeds grow with young erected shoots, which later become horizontal and produce long smooth stems bearing the leaves. *P. nigrescens* belongs to the Apocynaceae family, subfamily Periplocoideae, genus *Parquetina*. *P. nigrescens* is also known as *Periploca nigrescens* Afzel [11, 12], *Parquetina gabonica* Baill. [13], *Parquetina nigrescens* (Wennberg) Bullock [14], *Periploca afzelii* Don [15], *Periploca gabonica* (Baill.) [16, 17], *Periploca nigrescens* Wennberg [18], *Periploca preussii* Schum. [11], *Periploca wildemanii* Chev. Études) [19] and *Periploca calophylla* (Baill.) [20]. It is commonly found in African countries like Ghana, Nigeria, Cote d'Ivoire, Senegal, Zambia, Sudan, Zimbabody, and Angola [3, 8]. The local names for the plant vary from country to country. For instance, in Nigeria, the plant is called Ewe Ogbo, Mgbidim gbe, Kwankwanin, Inuwu elepe, and Ovie ukpakoma in tribes such as Yoruba, Igbo, Hausa, Yoruba (Ife), and Avianwu (Etsako), respectively [7, 10, 21]. In Ghana, the local name for the plant is Abakamo in the Twi dialect. *P. nigrescens* is usually found in regrowth forests and farmlands. Picking a leaf on the plant produces a milky fluid called latex, which becomes sticky when dried. Photographs of the full plant (Figure 11(a)), climbing stem (Figure 1(b)), upper surface leaf (Figure 1(c)), lower surface leaf (Figure 1(d)), flowering (Figure 2(a)), flowers on the plant (Figure 2(b)), fruits (Figure 3(a)), and seedlings (Figure 3(b)) have been displayed below.

### 3.2. Traditional Uses of *Parquetina nigrescens*

Traditionally, the leaf decoction of *P. nigrescens* mixed with a reasonable amount of honey is used in a bath to treat fatigue [22]. The same preparation is also used in the management of kidney problems, diabetes, insanity, diarrhea, jaundice, menstrual disorders, constipation, induced abortions, stomach ulcers [7, 9, 23, 24] anemia, asthma, and type 1 diabetes [25]. A decoction of the leaf in small portions is used to treat skin lesions, gastric ulcers, fever, and respiratory diseases in children [7]. Freshly crushed leaves are taken to prevent nausea and vomiting [23]. Leaf macerations using oil are applied to the legs of children having rickets and to the head to treat headaches [26]. The leaves are combined with *Cymbopogon citratus* and *Moringa oleifera* and boiled for the treatment of piles and lower back pain. A preparation of the dried leaves named (Tina-A tea) is currently used as herbal medicine for supporting the immune system, especially in asthmatics, at the Centre for Plant Medicine Research Mampong Akuapem, Ghana's outpatient clinic [27]. The roots are used for the treatment of menstrual pain and gonorrhea [5]. The root infusion is mixed with spices to treat measles, dysentery, intestinal worms, snake bites, and venereal diseases. The crushed roots are also mixed with olive oil or shea butter and applied to the hands or legs as snake and insect repellents [5]. A paste made from the roots is used for the treatment of convulsions in children. The decoction from the roots is used to treat nicotine poisoning [5]. The pulverized root bark, rubbed on the body, is considered a potent aphrodisiac [28].

The whole plant is used as medicine for the treatment of snakebite, wounds, piles, diabetes, as an aphrodisiac [29], and stress relief [30]. It is also taken to treat hypotension and ease childbirth. The decoction of the stem bark is used as a cardiac tonic [5]. The pulverized stem bark is applied to the skin surface to treat wounds and rheumatism. The leaf sap is used to treat jaundice and conjunctivitis [31]. The latex and leaf sap are externally used to treat burns and abscesses, applied to thorns in the skin to extract them [32]. The latex is used as an ingredient in arrow poison to hunt bush meat and is used to make black rubber [33]. The inner stem bark fibre is used in the manufacture of fishing nests and lines. It is also used to make ropes for tethering domestic animals and firewood [34]. The flowers of the plant are normally used for beautification and aesthetic purposes. The traditional uses of the plant parts and types of preparation are presented in Table 1.

### 3.3. Phytochemical Composition of *Parquetina nigrescens*

Phytochemicals are chemical compounds present in plants which are obtained through the secondary breakdown of the plants (metabolism). These compounds have biological activities in the plant and ensure the growth and development of the plant. It also protects the plant from pathogens and predators [35]. Phytochemical screening is imperative in detecting new sources of therapeutically and industrially important compounds. Previous phytochemical studies of the plant revealed that the ethanol extract of the leaves, roots, stem bark, and latex contained phytochemical compounds such as phenols, tannins, alkaloids, flavonoids, reducing sugars, phlobatannins, terpenoids, saponins, cardiac glycosides, steroids, and coumarin [9, 36]. Higher concentrations of alkaloids (0.0363 ± 0.0006%), saponins (0.0093 ± 0.006%), flavonoids (0.03 ± 0.004%), and tannins (16.5 ± 0.2%) were observed in ethanol leaf extraction of *P. nigrescens* when compared with Azadirachta indica with lower concentrations of alkaloids (0.0123 ± 0.0006%), saponin (0.0050 ± 0.0002%), flavonoids (0.02 ± 0.005%) and tannin (11.51 ± 0.385%) [3, 36]. Also, a quantitative investigation of the ethanol leaf extract of *P. nigrescens* revealed that it contained 2.5% saponins and 10.9% flavonoids [9]. In addition, the hydroethanolic leaf extract of *P. nigrescens* was found to contain alkaloids (72.00 ± 0.45%), saponins (18.00 ± 0.68%), tannins (4.12 ± 0.054%), cardiac glycosides (12.70 ± 0.12%), and anthraquinones (15.20 ± 0.34%) [37]. The presence of carbohydrates, unsaturated sterols, tannins, saponins, and phenolics has been reported in the methanolic stem extract of *P. nigrescens*, [38, 39]. Alkaloids, phenylpropanoids, terpenoids, anthraquinones, flavonoids, cardenolides, and saponins were found in the methanolic leaf extract of *P. nigrescens* [40]. An aqueous extract of the leaves contained alkaloids, iron, tannins, flavonoids, polyphenols, polyterpenes[41], anthraquinones, tannins, alkaloids, cardenolides, saponins, flavonoids, phlobatannis, ascorbic acid, and terpenoids [42–44]. Kayode and Yakubu [21] reported the presence of reducing sugar, cardiac glycosides, steroids, and terpenoids in aqueous leaf extract [45]. Isolation of compounds from methanol leaf extract revealed the presence of Alpha-phellandrene (1.726%), Naphthalene (0.750%), Catrienoate (0.884%), 5-ethyl-2-furaldehyde (2.412%), phytol (4.772%), squalene(7.990%), 7-hexadecyne (0.932%), Mannos (7.765%), 9-Octadecenamide (1.777%), Cymene (1.720%), 1,12-tridecadiene (0.981%), Alpha begarmotene (1.739%), Octadecanoic acid (0.646%), Beta-bisabolene (0.766%), Thiophene (6.814%), Octadecatrienoate (22.488%), Hexadecanoic acid (21.378%), decanoic acid (3.222%), Diethylphthalate (0.238%), Betacurcumene (0.742%), Octadecanoate(9.268%), and Pyranos (0.991%) [42]. The compounds phytol and squalene have been investigated in vivo for the treatment of renal damage in diabetic patients [33]. Volatile organic compounds isolated from the aqueous leaf extract of *P. nigresens* include 1, 2-benzenediol; 4H-pyran-4-one; 2, 3-dihydro-3,5-dihydroxy-6-methyl, and 5-hydroxymethylfurfural. These compounds have been found to produce cytogenotoxicity in some in-vitro studies [46].

### 3.4. Pharmacological Activities of *Parquetina nigrescens*

Pharmacological activities reported on *P. nigrescens* have been discussed below;

#### 3.4.1. Antianemic Activities of *P. nigrescens*

A study by Angèle and colleagues [41] found that an aqueous leaf extract of *P. nigrescens* administered by gavage at doses of 2000 mg/kg body weight and 2500 mg/kg body weight to phenylhydrazine-induced anemic rats (20 mg/kg/day phenylhydrazine hydrochloride) increased hemoglobin and the number of red blood cells while decreasing reticulocyte levels over the seven-day period of testing. The phenylhydrazine model of inducing anemia causes a decrease in haematological parameters such as red blood cells, hemoglobin level, and mean values of total bilirubin [41]. The extract showed a 99.73% and 64.91% recovery, respectively, for the 2500 mg/kg body weight and the 2000 mg/kg body weight doses, comparable to untreated anemic control rats (0.93%). The study discovered that 2500 mg/kg body weight of aqueous leaf extract provided a healthier recovery than the 2000 mg/kg body weight dose. Also, lower hemolysis of red blood cells was observed at both doses (0.003%), comparable to higher hemolysis of red blood cells (0.02%) for untreated (control) rats. This means that more young red blood cells were present in the treated anemic rats when the extract was administered than in untreated (control) rats. A higher osmotic resistance was observed in the treated anemic rats. The increase in the osmotic resistance of the red blood cells of the rats could be due to the presence of young cells. The administration of *P. nigrescens* extracts increased the production of young red blood cells. This could be attributed to the chemical compounds present in the plant that allow the recovery of young cells healthier than the blood cells after hemolysis. The finding showed that *P. nigrescens* could be used to treat anemia [41]. However, it can be observed that the experiment used very high doses, which may be difficult to replicate in human studies. Work using lower doses should be done for confirmatory purposes of the antianemic activity of the aqueous leaf extract. In alloxan monohydrate (5% w/v)-induced diabetic rats, normocytic and normochromic anemia were observed. Aqueous leaf extract of *P. nigrescens* administered at a dose of 1000 mg/kg body weight significantly increased hemoglobin, haematocrit, hemoglobin concentration, as well as mean corpuscular hemoglobin concentration. The extract reduced body and organ weight. This could be attributed to the lipid or cholesterol-reducing effect of the extract. The level of erythrocytes was reduced by depressing lipogenesis and osmotic fragility [25]. Previous research found that an aqueous leaf extract of the plant at a dose of 400, 800, and 1600 mg/kg body weight given to rats with acute blood loss increased hemoglobin concentration, reticulocyte count, red blood cell count, haematocrit, and erythrocyte indices [7, 47]. *P. nigrescens* aqueous root extract has also been investigated for haematological activity in Wistar rats. The results showed that the administration of the extract at doses of 50 mg/kg body weight increased mean red blood cell count, packed cell volume, and hemoglobin levels (6.72 ± 0.18, 43.0 ± 0.89, and 14.34 ± 0.29) when compared to the control (5.84 ± 0.22, 36.40 ± 0.87, and 12.12 ± 0.29) [26]. This could be attributed to the presence of phytochemicals such as flavonoids, tannins, and phenolic compounds present in the extract, which perhaps stimulated erythropoietin synthesis and hematopoietic effects. These findings reveal that the aqueous root extract of *P. nigrescens* at a minimal dose could be used in the treatment of anemia and may justify its use traditional ally in the treatment of anemia [26]. However, it is important that the doses used are checked for safety. Other models, such as the chemotherapy-induced anemia model, Heat-Killed Brucella Abortus (HKBA)-induced anemia model, levodopa-induced model, adenine-induced anemia model, aluminum-induced anemia model, and azidothymidine-induced anemia model, can be assessed to confirm the antianemic property of the various parts of the plant.

#### 3.4.2. Antidiabetic Activities of *P. nigrescens*

The antidiabetic activity of aqueous extract of the whole plant of *P. nigrescens* was evaluated in streptozotocin–nicotinamide-induced type 2 diabetic rats, administered at doses of 200, 400, and 800 mg/kg body weight. The results showed that the aqueous extract of the whole plant reduced blood glucose, *α*-glucosidase, and glucose-6-phosphatase activity and produced a significant increase in glycogen content and glucose tolerance [48]. *P. nigrescens* aqueous leaf extract was investigated for activity against diabetic mellitus in alloxan-induced diabetic (5% w/v) Wistar rats. Alloxan was administered intraperitoneally at a dosage of 100 mg/kg body weight, and the aqueous leaf extract was also administered at 1000 mg/kg body weight. The result showed that the aqueous leaf extract significantly reduced the blood glucose level (5.52 ± 0.55) and complete blood count with a comparable effect to the dosage of 25 mg/kg body weight of chlorpropamide (6.90 ± 0.41) and alloxan (16.46 ± 2.70) consistently for a period of 4 weeks [25]. A methanol leaf extract of *P. nigrescens* was also evaluated for renoprotective effects in alloxan monohydrate-indued diabetic (120 mg/kg) Wistar rats. At doses of 100 mg/kg body weight and 200 mg/kg body weight, the 200 mg/kg body weight dose significantly increased catalase and glucose 6-phosphate dehydrogenase, glutathione peroxidase, lactate dehydrogenase, superoxide dismutase, and albumin levels in the diabetic treated rats, compared with the diabetic normal or untreated rats (0.3 ml distilled water administration). Reduced creatinine and blood urea nitrogen were observed at the same dose in diabetics treated and untreated (0.3 ml distilled water) in comparison with increases in creatinine and blood urea nitrogen in diabetics untreated [42]. Creatinine is the bioproduct obtained through metabolism from muscle creatine and expelled via the kidney [49]. The significant reduction in the creatine level in the treated rats indicates normal kidney function, anti-inflammatory, and increased glomerular filtration. In another antidiabetic study, the methanol leaf extract of *P. nigrescens* administered at doses of 1000 mg/kg and 2000 mg/kg had significant decreases in fasting blood glucose when compared to the diabetic untreated group at weeks 3 and 4 of administration. Liver glycogen levels were significantly decreased at a dose of 1000 mg/kg·p.o. A decrease in duodenal glucose absorption was observed at 20 and 60 minutes in rats treated with 1000 mg/kg and 2000 mg/kg·p.o. Serum glucose levels significantly decreased in the treated group. The insulin concentrations increased in extract treated rats compared to untreated rats. The decrease in blood glucose could be attributed to the bioactive compounds such as squalene, phytol, and the ability of the methanol leaf extract to increase insulin production necessary for glucose usage [33, 42]. Though this work has shown the ability of the extracts to decrease blood glucose levels, the doses used are relatively too high and may be impractical to administer in humans and/or may cause toxicity problems. More studies are needed to ascertain the minimum doses at which antidiabetic activity may be obtained in addition to its toxicological profile. Adeyomoye and colleagues conducted a study where methanol leaf extract of *P. nigrescens* was administered at a dose of 100 and 200 mg/kg body weight in alloxan-induced diabetic rats (120 mg/kg) for 28 days [33]. They suggested that phytol and squalene isolated from the methanol leaf extract may be responsible for the improvement in the function of the kidney in diabetic mellitus possibly by increasing urea synthesis in urine metabolism and increasing the excretion of urea. Faloye and colleagues [50] conducted research on the methanol root bark extract of *P. nigrescens* and the convallatoxin compound isolated from *P. nigrescens* root bark. Administration of the methanolic root extract to streptozotocin-induced diabetic rats at a dose of 200 mg/kg body weight showed a significant dose and time-dependent reduction in blood glucose levels that was comparable to the standard control glibenclamide at 5 mg/kg for 4 hours. In addition, the methanol extract reduced the blood glucose level by 70% s in 10 days, compared with a 39% reduction of blood glucose by glibenclamide in the same days. The ethyl acetate fraction of the root extract contained the isolated compound convallatoxin. Convallatoxin (20 mg/kg) was administered to streptozotocin-induced diabetic rats. The result showed a time-dependent reduction in blood glucose levels with 35% for convallatoxin and 38% for glibenclamide at 4 hours. Convallatoxin reduced blood glucose by 83% on day 10, comparable to 38% for glibenclamide. This research validates the use of *P. nigrescens* in the treatment of diabetes. However, checks at more dosage points are needed for confirmatory purposes in addition to the possible toxicological profile of the compound convallatoxin at the studied dose. A polyherbal consisting of *P. nigrescens* and *Erythrina senegalensis* has been reported to have the potential to treat diabetes. The study used streptozotocin-induced models in Wistar rats at doses of 65 mg/kg body weight and nicotinamide at a dose of 230 mg/kg body weight. The aqueous leaf extract of *P. nigrescens* and *Erythrina senegalensis* was administered at doses of 400, 600, and 800 mg/kg body weight. The result from the study showed that at doses greater than or equal to 600 mg/kg body weight of the drug, the level of hyperglycemia was significantly reduced in a dose-dependent manner in treated rats. The levels of total cholesterol and triglycerides also significantly decreased after 28 days. A significant decrease in glycated hemoglobin at doses of 800 mg/kg body weight after 90 days was observed. The study showed that leaf extracts of *P. nigrescens* and *Erythrina senegalensis* have anti-hyperglycemic and antidiabetic properties [51]. In this study, it can be observed that the doses used are relatively high; hence, more work is needed to support the use of the plant in the treatment of diabetes in humans. In addition, due to the fact that diabetes is a chronic condition which requires continuous medical treatment, more studies on chronic toxicity are needed to justify its safe use at practical doses in humans.

#### 3.4.3. Antihyperlipidemic Activities of *P. nigrescens*

The use of the aqueous extracts of the whole plant of *P. nigrescens* for their antihyperlipidemic effect in traditional medicine has been supported by a study by Ojuade and colleagues [48]. The antihyperlipidemic effect of the aqueous extract of the whole plant was investigated using streptozotocin-nicotinamide-induced type 2 diabetic rat models. In the study, serum lipase, low-density lipoproteins, total cholesterol, total triglyceride, atherogenic index, coronary risk index, pancreatic-amylase, and lipase activities were all reduced after treatment with aqueous extracts (400 and 800 mg/kg) of the whole plant for 14 days. Significant increases in glutathione, superoxide dismutase, catalase, high-density lipoproteins, adiponectin, and leptin were observed in addition to the recovery of pancreatic tissues [48]. An aqueous root extract of *P. nigresecens* was investigated for antilipid activity in albino rats. The results revealed that a dose administered at 100 mg/kg body weight caused an increase in mean total cholesterol levels, low-density lipoproteins, and no alteration in high-density lipoprotein after 21 days. However, extract administered at a dose of 150 mg/kg showed a significant increase in mean total cholesterol, triglycerides, low-density lipoprotein, and high-density lipoprotein when compared to the control (10 ml/kg of normal saline). The variation in lipid concentration could be explained by lipid metabolism, which causes the heart to accumulate fats, cholesterol, and other substances in the arteries [26]. Moreso, an increase in these lipid profiles such as low-density lipoprotein, cholesterol, and high-density lipoprotein of the aqueous root extract at doses of 100 and 150 mg/kg body weight of *P. nigrescens* in the Wistar rats may be a beneficial index in examining the effect of these extracts on the metabolism of lipids and how humans and animals may be vulnerable to coronary artery diseases from consumption of preparations from these plants [26]. These findings call for more studies into the lipid regulating abilities using varying models of various extracts obtained from different parts of the plant to help inform consumers about any potential danger that may exist as a result of consumption of the plant.

#### 3.4.4. Antioxidant Activity of *P. nigrescens*

Antioxidant activity was investigated using aqueous, methanol, and flavonoids isolated from methanol leaves and stem extracts of *P. nigrescens* in Wistar rats. The scavenging effect, reducing power, and inhibition of Fe2+/ascorbate-induced mitochondrial lipid peroxidation in the rat liver were investigated, respectively. The result showed a significant increase in the scavenged 2,2-diphenyl-1-picrylhydrazyl generated radicals at 1000 *μ*g/ml of the flavonoid, being the highest followed by methanol and aqueous leaf and stem extract of *P. nigrescens.* Also, a similar paradigm was observed with the extract at 1000 *μ*g/ml for reducing power. At 50 mg/ml of *P. nigrescens* leaves and stems extract, the Fe2+/ascorbate-induced lipid peroxidation in rat liver mitochondria possessed significant inhibitory effects. The results from the study showed that different extracts from the leaf and stems of *P. nigrescens* possessed antioxidants. This could be valuable in reducing superoxide generation reactions in the body and may be used in the treatment of liver diseases [3]. Akinrinmade and colleagues [52] investigated the defensive potential of methanol leaves extract of P. nigrescens against ischemia-reperfusion injury in the intestines of Wistar rats. The methanol extracts were administered at doses of 500 mg/kg and 1000 mg/kg. The results showed a significant decrease in the intestinal epithelial lesions (inflammation, villi erosion, and hemorrhage) comparable to ischemia-reperfusion injury. The treatment of methanol leaf extract prevented escalations in malondialdehyde, glutathione S-transferase, and glutathione. A considerable alleviation of intestinal injury produced by ischemia-reperfusion was also observed. Oghenejoboh and Nkop [53] used a 2, 2-diphenylpicryl hydrazine (DPPH) radical scavenging assay to demonstrate the antioxidant activity of essential oil from the leaves and stem of *P. nigrescens*. The essential oil with concentrations of (1.0, 0.5, and 0.25 mg/mL) showed better scavenging of free radicals when compared to standard antioxidants butylated hydroxyl anisole administered at a dose of 1.0 mg/mL. The antioxidant potential of the essential oils showed that it may be used for the formulation of drugs and creams for the treatment of inflammatory conditions since most are caused by reactive oxygen species. The finding showed that increases in the concentration of the extract led to an increase in the scavenging potential of *α*, *α*-diphenyl-*β*-picrylhydrazyl (DPPH) radicals [36]. This may be attributed to the radical scavenging activity of phenolic compounds present in the plant. An ethanolic leaf extract of *P. nigrescens* was investigated for activity in myocardial infarction in albino rats using an isoproterenol-induced model (85 mg/kg body weight) for 14 days. The extract was administered at doses of 100 and 200 mg/kg body weight. The result showed a reduction in antioxidant parameters such as glutathione reductase, superoxide dismutase, and catalase activities of the heart when compared to increases in the same parameters in the serum of isoproterenol-inducedmyocardial-infracted rats. Furthermore, decreases in the heart function indices such as cardiac troponin, creatine kinase, sodium potassium ATPase, and lactose dehydrogenase in the rats administered with ethanolic leaf extract of *P. nigrescens* were observed. There were decreases in inflammatory markers such as interleukin 1*β*, tumour necrotic factor, and interleukin 6 when compared to isoproterenol-induced myocardial infarction rats. There was no adverse effect of histopathological studies on the rats, and normal cell architecture was observed [54]. A study by Ademola and colleagues [28] showed the potential of the polyphenol-rich fraction of *P. nigrescens* to mitigate dichlorvos-induced cardiorenal toxicity in a rat model. The result showed that the polyphenol-rich fraction administered at doses of 100 and 200 mg/kg reduced cardiac nitrotyrosine and mitogen active protein kinase in the rats, exhibiting protective properties in the heart and kidney of the rats exposed to dichlorvos.

#### 3.4.5. Antiulcerogenic Activity of *P. nigrescens*

Hexane and chloroform leaf extracts of *P. nigrescens* were investigated at an administered dose of 500 mg/kg body weight and 1000 mg/kg weight using ethanol-induced ulcer models in rats. Also, the activity of superoxide dismutase, catalase, and levels of reduced glutathione were evaluated in the gastric mucosa and liver of normal and experimental groups of rats. The result showed that ethanol-induced gastric ulcer was significantly reduced with the hexane and chloroform extracts of *P. nigrescenes*. The activities of superoxide dismutase, glutathione, and catalase were lower as compared to the normal control saline [55]. Meanwhile, there was a significant reduction in the activity of superoxide dismutase in the ulcerated mucosa comparable to the normal control. There were significant increases in catalase and glutathione in the extract treated group with the extract compared to the ethanol-induced group. Pretreatment with the extract of *P. nigrescens* showed gastro-protective and anti-ulcer effects. This could be attributed to the stimulation of antioxidant enzymes providing defense against ethanol-induced ulcer effects [55]. Aqueous leaf extract of *P. nigrescens* was investigated for gastrointestinal protective potential in adult male albino rats using ethanol-induced gastric ulceration. The results showed that the aqueous leaf extract of *P. nigrescens* at doses of 100 and 500 mg/kg body weight significantly decreased gastric-acid secretion and caused an increase in gastric mucus secretion relative to the normal control group (water administered) [5]. The decrease in gastric-acid secretion in animals pretreated with the extract shows that the extract could be considered as a potential substitute for antisecretory agent standard drugs such as cimetidine or ranitidine. Also, the extract causing an increase in gastric mucus secretion could be likened to carbenoxolone sodium [5]. From this study, it can be observed that the doses used are relatively high and few, more dose points using other antiulcer models may help to confirm this pharmacological activity. The mechanism(s) of antiulcerogenic action should also be investigated for confirmation.

#### 3.4.6. Antisickling Activity of *P. nigrescens*

The hydro-methanolic extract obtained from a combination of leaves and stems of *P. nigrescens* has been evaluated for antisickling activity, erythrocyte membrane-stabilizing effects, and organ toxicity at a dose of 0–5 mg/mL of the extracts in albino rats. The percentage reversal and inhibition of sickling parameters were analyzed on presickled Sickle hemoglobin (Hb^SS^) blood cell suspensions using 2% sodium metabisulphite solution as an inducer, with parahydroxybenzoic acid and normal saline as positive and negative controls, respectively [23]. The effects of plant extracts on erythrocytes were assessed using osmotic fragility, and the toxicity profile was determined using LD50 and subacute toxicity studies on graded extract concentrations. The results showed that the extract of *P. nigrescen* has significant antisickling activity. The sickle hemoglobin (Hb^SS^) was reduced to 5% at 40 min, compared with the sickle hemoglobin (Hb^SS^) control (parahyrodroxyl benzoic acid and normal saline), which had over 65% sickled cells at the tested dose. No toxicity was observed at the tested dose. The stability of the erythrocyte membrane was protected as observed by the decrease in percentage hemolysis of the sickle hemoglobin (Hb^ss^) at the 5 mg/mL dose of the extract using a 0.25% buffered saline concentration [23]. The mechanism for antisickling activity may be the extension of the interval time of hemoglobin polymerization [23]. Further studies should be conducted using prolonged time and varied doses of the plant. Other parts of the plant should also be investigated for anti-sickling activities. Kade and colleagues [56] have also reported the antisickling activity of the root extract of *P. nigrescens*, indicating that using the combination of leaves, stems, and roots may be more effective as a healing agent in the treatment of sickle cell anemia. Kplé and colleagues [57] investigated the antisickling activity of hydroethanolic extracts from *P. nigrescens* and a combination product (*Justicia secunda*, *Jatropha gossypiifolia*, and *Parquetina nigrescens*) in a ratio of 1 : 1 : 1 using a reversal test. The Hb SS blood sickling was induced with 2% sodium metabisulphite. The result showed that the polyherbal with an extract concentration of 10 mg/mL had a higher percentage of sickling reversal effect (75.00 ± 4.33%) when compared to the negative control (10.17 ± 0.55%). Also, the hydroethanolic extract of *P. nigrescens* at a concentration of 10 mg/mL had a percentage sickling reversal effect of (77.83 ± 2.89%) when compared to the negative control (10.17 ± 0.55%). This could be attributed to the phenolic compounds and phenylalanine present in the plant [58]. More studies are needed on *in-vivo* antisickling using different models such as hypoxia-induced models. In addition, the dosing points may be varied in further studies and their toxicity (especially chronic) also be checked considering the chronic nature of sickle cell disease. These antisickling studies may justify the use of the plant in the treatment of sickle cell anemia.

#### 3.4.7. Antimalarial Activity of *P. nigrescens*

Nafiu and colleagues [59] reported an in-vivo study of the antimalarial potential of aqueous leaf extract of *P. nigrecsens* and a combination of aqueous leaf extract of *P. nigrescens* and Tithonia diversifolia in albino mice. The result showed that the aqueous leaf extract of *P. nigrescens* administered at a dose of 150 mg/kg body weight showed good inhibition with an 86% reduction in the parasitaemia by chemo-suppression for 18 days against the malaria parasite (*Plasmodium berghei*) in albino mice. The combined extracts (*P. nigrescens* + *T. diversifolia*) demonstrated significant antimalarial activity and 90% parasite inhibition at administered doses of 150 mg/kg body weight. The mean survival time for mice recorded was 19 days for the combined extracts, and the aqueous leaf extract of *P. nigrescens* was 18 days when compared to 7 days for the control (0.3 ml of distilled water). Studies have reported that the survival of experimental animals beyond 12 days of malarial infection is regarded as an activity [60, 61]. The use of aqueous leaf extracts separately or in combination could be a possible alternative for the treatment of malaria [60] More *in-vivo* antimalarial and toxicological studies may be needed to ascertain the efficacious and safe doses of the extracts. Other animal and human models such as *Plasmodium yoelii*, engraftment of hematopoietic stem, and progenitor cells (HSPCs) into immune-deficient mice and use of humanized mice should be used to confirm the antimalarial properties of the plant. Also, parts of the plant such as the stem and roots should also be assessed for potential antimalarial activity.

#### 3.4.8. Antidiarrhoeal Activity of *P. nigrescens*

Mahmud and colleagues [34] conducted an *in-vivo* study with methanol root extract of *P. nigrescens* for the treatment of diarrhoea using castor oil-induced diarrhoea, castor oil-induced enteropooling, and a gastrointestinal motility assay. The findings from the study revealed that the methanol root extract of *P. nigrescens* administered at the doses of 25, 50, and 100 mg/kg significantly interrupted the inception of diarrhoea by inhibiting the production of wet feces. Also, the occurrence of defecation was reduced significantly at doses of 50 mg/kg and 100 mg/kg. The methanol root extract decreased the volume of intestinal fluid accumulated at all doses in the castor oil-induced enteropooling assay. The peristalsis index was reduced when the methanol root extract was administered at doses of 25, 50, and 100 mg/kg for the gastrointestinal motility assay. The study validates the use of the methanol extract of *P. nigrescens* in the treatment of diarrhoea. In another study, *in-vivo*anti-diarrheal activity was investigated using *E. coli* and castor oil-induced diarrhea models. A mixture of methanol leaf and stem bark extract of *P. nigrescens* and *Parkia biglobosa* at doses of 600 mg/kg body weight gave significant inhibition of defecation, with a 71.42% inhibition in castor oil-induced diarrhea models [40]. The combination extract used in the castor oil-induced diarrhea showed more helpfulness in controlling diarrhea than the individual plants at doses of 600 mg/kg body weight. The extract probably prevents diarrhea by inhibiting the production of prostaglandins through the stimulation of the epithelial cells, hence preventing the release of nitric oxide and adenylyl cyclize in the castor oil-induced diarrhea assay [62]. However, the Methanol leaf extract of *P. nigrescens* in both *E. coli* and castor oil-induced diarrhea had better alkaline phosphatase, aspartate transaminase, and alanine transaminase levels relative to the negative control [40]. More doses of the extract with different plant parts are needed to confirm its mechanism of managing diarrhoea.

Hydro–methanolic extract of *P. nigrescens* leaves and a stable suspension formulated from them (1.25% w/v to formulate a structured vehicle (carboxymethylcellulose, polyvinylpyrrolidone, and tragacanth) were investigated for antidiarrheal activity using castor oil-induced diarrhea in Wistar rats. The extract was administered at doses of 5 mg/kg and 200 mg/kg body weight. The result showed that in castor oil-induced diarrhea rats, a dose-dependent decrease in the frequency of watery stools was observed with percentages of 35.29% and 64.70%, respectively. There was a significant reduction in intestinal transit of charcoal meal by 0.14% and 0.15% at doses of 5 mg/kg body weight and 200 mg/kg body weight when compared to a 0.12% inhibition by 5 mg/kg body weight of the control (loperamide). The reduction in the intestinal transit time reveals that the extract reduces the motility of the gut, hence reducing diarrhea. The antidiarrheal effects of the suspension at doses of 5 mg/kg and 200 mg/kg were also dose-dependent and lasted for 4 weeks. Both the extract and the suspension had substantial antidiarrheal activity [63]. Varying doses of both extract and suspension within the range of 5 mg/kg to 200 mg/kg body weight should be tested to help develop optimized doses for consideration in the treatment of diarrhoea. Other considerations of possible toxicity should also be investigated due to the solvent system used. Isolated compounds from the extract may also be further investigated to ascertain antidiarrhoea activity, which may possibly lead to the development of novel drugs.

#### 3.4.9. Anti-inflammatory, Anti-pyretic, and Analgesic Activity of *P. nigrescens*

Aqueous leaf extract of *P. nigrescens* was evaluated for analgesic, anti-inflammatory, and antipyretic activity in rats. Formalin paw licking and hot plate latency assays for analgesic activity were used. The cotton pellet granuloma, carrageenan oedema, and formaldehyde arthritis models were used to investigate anti-inflammatory activity, while the brewer's yeast test was used for antipyretic activity in the rats. The result showed that the aqueous leaf extract at the dose of 200 mg/kg body weight at a reaction time of 90 mins produced significant pain relief (4.30 ± 0.25%) by increasing the latency period in the hot plate assay when compared to the control saline (2.90 ± 0.16%) and to the reference indomethacin at a dose of *5 mg/kg body weight* (3.8 ± 0.12%). Similarly, in formalin paw licking, the aqueous leaf extract reduced the paw licking significantly at the doses of 50 and 200 mg/kg at the later phase (31.2 ± 2.9% and 33.2 ± 23.3%) in comparison to control saline (87.4 ± 4.4%), thus the extract exhibits significant analgesic activity. These findings support the local use of the decoction of the plant for the treatment of headaches [64]. In addition, the administration of the aqueous leaf extract reduced the carrageenan-induced paw oedema in formaldehyde-induced arthritis models at doses of 50 and 200 mg/kg body weight as the days prolonged when compared to control saline, which observed an increase in the paw size. In cotton pellet granuloma induced models, 45.4% inhibition of granuloma tissue formation was observed with the aqueous leave extract at a dose of 200 mg/kg body weight, compared to zero (0%) inhibition with the control (saline). The arthritic limbs were immobilized in a pattern similar to that of indomethacin when compared to the control (saline). In the brewer's yeast-induced models, the extract also inhibited the pyrexia induced by brewer's yeast at a dose of 200 mg/kg when compared to the control. These studies on the aqueous leaf extract of *P. nigrescens* validate traditional uses of the plant in the treatment of inflammation, pain-related disorders, and fever [64]. Methanol fruit bark extract of *P. nigrescens* was evaluated for antinociceptive effects using formalin and acetic acid-induced pain at doses of 50 mg/kg, 100 mg/kg, and 200 mg/kg in Swiss albino mice. 5% Tween 80 at 10 ml/kg and 100 mg/kg body weight of acetylsalicylic acid were used as controls. The results showed significant dose-dependent antinociceptive potential at doses of 50–200 mg/kg body weight at both the early and late phases of inhibition. At doses of 200 mg/kg body weight, the highest percentage inhibition observed was (57.18%) in the early phase and (78.68%) in the late phase, which is comparable to the positive control acetylsalicylic acid at a dose of 100 mg/kg body weight with 80.15% inhibition [65]. The extract possibly inhibits both the peripheral and central pain mechanisms in the animal models. A similar finding was reported by Owoyele and colleagues [64] on the aqueous leaf extract of *P. nigrescens*, which showed a significant antinociceptive effect at doses of 50–200 mg/kg body weight in a formalin-induced model. The methanol fruit bark extract of *P. nigrescens* showed writhing inhibition (52.31%) in an acetic acid-induced model at a dose of 200 mg/kg body weight compared to the positive control acetylsalicylic acid (85.64%). A higher percentage of writhing inhibition was observed for acetylsalicylic acid in the acetic acid-induced model. The extract at doses of 50–200 mg/kg body weight also inhibited the writhing response for 30 mins in the mice. This could be attributed to the fact that the mechanism of analgesia of the extract may be a combination of both central and peripheral mechanisms and not only inhibition of prostaglandins [65] The potential of the extracts from the leaves and fruit barks needs to be further investigated for efficacy and safety in the management of pain-related conditions. Other parts of the plant, such as the stem and roots, may also be investigated.

#### 3.4.10. Antiasthmatic Activity of *P. nigrescens*

Aqueous leaf extract of *P. nigrescens* produced a fall in the contractile effect of histamine as well as carbachol on the trachea of guinea-pigs. The doses ranged from 0.024 to 1.536 mg/mL [66] showed that 400 mg/kg body weight and 800 mg/kg body weight of the extract showed defensive effects against histamine and antigen-induced broncho spasms when compared with the standard drug, astemizole, at a dose of 5 mg/kg body weight [66]. More studies, especially clinical observational and trials on the antiasthmatic activity of various parts of the plant, must be conducted to justify its use in the treatment of asthma. More *in-vivo* antiasthmatic models such as the Ovalbumin (OVA)-sensitized murine model may also be investigated.

#### 3.4.11. Toxicological Activity of *P. nigrescens*

The toxicity of a given plant is determined by factors such as genetic differences between species, plant parts used, climatic conditions, type of soil and location of the plant, the potential of the phytochemical and bioactive compounds isolated, the quantity of extracts or the dose regimes of the extracts consumed, and individual body chemistry [67]. Aqueous leaf extract of *P. nigrescens* was investigated for subacute toxicity using the brine shrimp lethality bioassay and Lorke's method in male mice at doses of 2000, 4000, and 8000 mg/kg body weight. The result showed no signs of toxicity or mortality detected among the treated mice throughout the 28-day period of the administration [43]. However, there were changes in the histomorphology of the vital organs, including mild kidney interstitial fibrosis, mild liver microhemorrhages, and mild glomerular hypercellularity of the aqueous leaf extract at a dose of 8000 mg/kg body weight. The treatment with aqueous leaf extracts for 28 days significantly decreased hemoglobin, packed cell volume, and red blood cell count. Biochemical parameters such as transaminases are known as good indicators of liver function and are used as biomarkers to anticipate the toxicities of drugs. The increase in enzyme transaminases in the blood occurs as a result of damage to the liver parenchymal cells. The aqueous leaf extracts of *P. nigrescens* significantly increased alanine aminotransferase and aspartate aminotransferase at a dose of 2000 mg/kg body weight. Meanwhile, at a dose of 8000 mg/kg body weight, the alanine aminotransferase, aspartate aminotransferase, potassium, and triglyceride levels were significantly reduced. The aqueous leaf extract of *P. nigrescens* administered at the various doses did not reveal any end organ toxicity when assessed by histological and biochemical analysis, making it moderately safe as a medicinal plant [43]. However, there is a need to advise against the use of excessively high doses of the plant. In Sprague–Dawley rats, the toxicological activities of powdered leaves and aqueous extract of *P. nigrescens* were investigated for sub-chronic activities. The extract was administered at a dose of 400, 800, and 1600 mg/kg·p.o for six weeks. The result showed that the extract did not cause any major significant change in the biochemical parameters such as alanine aminotransferase and aspartate aminotransferase. The enzyme levels remained unchanged. The histological parameters, including red blood count, had no significant changes. The mean serum urea levels remained statistically unchanged over the six-week duration of the treatment with the extract in the rat. The liver and kidney did not experience any recorded toxic effects or changes [27]. An acute toxicity study was reported using methanol leaf extract of *P. nigrescens* in diabetic Wistar rats at doses of 100 mg/kg and 200 mg/kg. The result showed no cases of mortality in the various treated Wistar rats [42]. The methanol leaf and aerial part extract, administered at a dose of 1000 mg/kg body weight, exhibited restoration of glomerular tufts, improvement of vasculature and liver, as well as a reduction in inflammatory infiltration with healthy hepatocytes in subchronic toxicity [68]. In addition, there was an increase in the mean count of haematological parameters such as red blood cells, hemoglobin, and haematocrit concentrations when compared with the control group (distilled water). Generally, it can be observed that more studies on the toxicity (acute, subchronic, and chronic) of the various parts of the plant are needed as it is used for the management of several diseases, most of which require long periods of usage. The minimum safe dose of various extracts from various parts of the plant should also be established using appropriate toxicological studies.

*Cytotoxic activity of P. nigrescens.* According to Onyegeme–Okerenta and colleagues [69], an ethanol leaf extract of *P. nigrescens* demonstrated significant cytotoxic potential in a dose-dependent manner, with cytotoxicity increasing with an increase in the concentration of the extract in human cancer cell lines. These cell lines were: MCF-7 (breast carcinoma cells), C4-2WT (prostate carcinoma cells), HCT 29 and HCT 116 (colorectal carcinoma cells) using (3-(4, 5-dimethylthiazol-2-yl)-2,5-diphenyltetrazolium bromide), methylene blue proliferative assay and trypan blue assay for cell count. In the (3-(4, 5-dimethylthiazol-2-yl)-2,5-diphenyltetrazolium bromide (MTT) assay, the growth inhibition of the extract of *P. nigrescens* required for 50% inhibition of the four cell lines was below the 30 *μ*g/ml criteria postulated by the American National Cancer Institute. The percentage inhibition of the cells was MCF-7 = 2.61 *μ*g/ml, C4-2WT = 8.33 *μ*g/ml, HCT 29 = 3.47 *μ*g/ml, and HCT 116 = 1.75 *μ*g/ml for 72 hours. An increase in the concentration (0.1 to 100 *μ*g/mL) of the *P. nigrescens* extract increased the cytotoxicity potential of the four human cancer cell lines when compared to control cells (untreated). Moreso, in the methylene blue assay, increases in the concentration of the extract and the time of exposure of the cells to the extract reduced the number of viable cells. Also, in the trypan blue assay, the post-treatment of the ethanol leaf extract of *P. nigrescens* at a dose of (10, 20, 30, and 40 *μ*g/mL) for a duration of 72 hours reduced the total viable cells. Meanwhile, total nonviable cells were increased, which could be due to the cytotoxicity potential of the extract [69]. A moderate cytotoxic potential was observed when essential oil obtained from the leaves and stem of *P. nigrescens* was investigated using a brine shrimp lethality assay. The result showed that the essential oils administered at doses of 10, 100, and 1000 ppm had LC50 values (lethal concentration at 50%) a concentration of fewer than 100 *μ*g/mL thus, the lethal concentration of leaf oil and stem oils was 14.90 and 38.04 *μ*g/mL respectively, representing moderate cytotoxic activity. The oils could be useful in developing drugs for the treatment of diseases caused by abnormal cell growth and reactive oxygen species [53]. Methanol leaf and aerial part extract of *P. nigrescens* were investigated in Wistar rats for cytotoxicity. The influence of extracts on the release of lactase dehydrogenase from HaCaT keratinocytes cells was evaluated with a concentration of 0.1 to 100 *μ*g/mL of the extracts. The result showed a significant increase in the amount of lactase dehydrogenase released from HaCaT keratinocytes cells in comparison to untreated cells (negative control) [68]. Further studies of the cytotoxic activity of the extracts will help to justify its usefulness in the treatment of cancers.

*Cytogenotoxicity activity of P. nigrescens.* Aqueous leaf extract of *P. nigrescens* has been investigated by Alabi and colleagues [46] on the root meristematic cells of Allium cepa for cytotoxic and genotoxic effects. Allium cepa was exposed to extract concentrations ranging from 1, 5, 10, 20, and 50%. The results showed that at a concentration of 5 to 50%, cytotoxicity was observed by inhibition of root growth as compared to the control (lead nitrate at 10 ppm). Moreover, extracts at concentrations of 10% and 20% showed a significant reduction in chromosomal abnormalities, thus demonstrating genotoxicity. Active compounds such as 1, 2-benzenediol, 2, 3-dihydro-3,5-dihydroxy-6-methyl 5- hydroxymethylfurfural, and 4H-pyran-4-one were supposed to be liable for the detected cytogenotoxicity. The consumption of the aqueous crude leaf extracts of *P. nigrescens* at high concentration could be harmful, so care should be taken since the high concentration of the leaf extract resulted in cytogenotoxicity. Since cytogenotoxicity was only studied in a plant model on *P. nigrescens,* a study of cytogenotoxicity in human cells (MCF-7 human breast adenocarcinoma cell) and animal species based on micronuclei formation using bone marrow micronuclei test, peripheral blood erythrocyte micronuclei test, and lymphocyte cytokinesis blocking micronuclei assay is required.

*Anti-neurotoxicity activity of P. nigrescens*. A hydroethanolic leaf extract of *P. nigrescens* was evaluated against D-galactose-induced (300 mg/kg body weight) neurotoxicity in male Wistar rats. The results showed that the administration of the extract at doses of 250 and 500 mg/kg body decreased 8-malondialdehyde levels and increased the activities of superoxide dismutase and catalase. As well as improved glutathione concentration were observed. The extract also improved the activities of brain caspase 3-dependent apoptosis, acetylcholinesterase, tumor necrosis factor *α*, and DNA fragmentation index. Provision of neuroprotection against D-galactose-induced brain damage through antioxidative, anti-inflammatory, as well as antiapoptotic mechanisms may have occurred. These protective effects could be as a result of a profile of phytochemicals such as flavonoids (quercetin and luteolin), alkaloids, and phenol present in the leaves of the plant [70]. Testing at more doses (especially lower doses) should be carried out. Other parts of the plant should also be studied for their neurotoxic effects.

### 3.5. Antimicrobial Activity of *P. nigrescens*

Aqueous leaf and ethanol extract of *P. nigrescens* were investigated against some strains of Gram-positive and Gram-negative bacteria, including *Staphylococcus aureus*, *Salmonella typhi*, *Proteus mirabilis*, *Pseudomonas aeruginosa*, *Bacillus* subtilis, and *Proteus vulgaris*. The result showed that the aqueous leaf extract showed antimicrobial potency against all the above microorganisms with minimum inhibitory concentrations (MICs) of (3, 6, 9, 10.5, 15, and 18 *μ*g/mL), respectively. Meanwhile, the ethanol leaf extract was only effective against *Pseudomonas aeruginosa* and *Salmonella typhi* with a minimum inhibitory concentration of 15 *μ*g/mL. These reported MICs give potential for use of the aqueous leaf extract of the plant as an alternative antibiotic agent looking at its broad spectrum of action. In an *in-vivo* study conducted by Oloyede and colleagues [71], the intestinal microbial count (CFU/g) of caecum samples from rats administered with a concentration of 50 mg/kg body weight of both aqueous and ethanol leaf extract was evaluated. The result showed no reduction in the total bacteria count and total lactobacilli count, having a mean bacteria count of 95.00 ± 8.145 CFU/g and 94.33 ± 2.906 CFU/g when compared to the control (standard chow and water), 98.33 ± 9.528 CFU/g and 1.33 ± 1.886 CFU/g. This implies that the aqueous and ethanol leaf extract of *P. nigrescens* could not affect beneficial microbes like lactic acid bacteria in the gastrointestinal tract that might lead to imbalances within the gastrointestinal tract [72]. In an in-vitro study, the ethanol leaf extract showed the highest (90%) inhibitory potential at all the doses (50, 100, and 200 mg/kg body weight) on isolated microorganisms such as *Proteus vulgaris*, *Cirobacter aerogenes*, *Pseudomonas fluorescens*, *Klebsiella oxytoca*, *Lactobacillus acidophilus*, *Lactobacillus alimentarus*, *Lactobacillus plantarum*, and *Lactobacillus fermentum* from the gut of Wistar rats, compared to the lowest inhibitory potential of 30% observed when 50 mg/kg aqueous extract was administered [71]. In another study, aqueous and ethanol leaf extracts of *P. nigrescens* were investigated *in-vivo* in Wistar rats for gastrointestinal microflora. The mean total bacteria count revealed a significant reduction in *Enterobacteriaceae* and *Lactobacilli* with aqueous leaf extracts administered at a dose of 100 and 200 mg/kg body weight, having a mean total *Enterobacteriaceae* count of (41.67 ± 1.764 CFUg and 34.33 ± 2.333 CFU/g) and a mean total *Lactobacilli* count of (40.67 ± 3.528 and 35.00 ± 2.646a CFU/g). Also, ethanol extract administered at a dose of 100 and 200 mg/kg body weight had a mean total *Enterobacteriaceae* count of (34.00 ± 1.155 CFU/g and 25.33 ± 1.200 CFU/g) and a mean total *Lactobacilli* count of (37.33 ± 2.728 CFU/g and 19.33 ± 0.88 CFU/g), respectively, when compared to the control (standard chow and water) (57.33 ± 1.764 CFU/g and 71.33 ± 1.886 CFU/g). This implies that the higher the concentration of aqueous or ethanol leaf extract administered, the greater the reduction in the microbial load in the *in-vivo* study. Higher concentrations (100 mg/kg and 200 mg/kg body weight) should be used with caution because the reduction effect may lead to gastrointestinal tract imbalance because beneficial microbes such as lactic acid bacteria play a role in the gastrointestinal tract [72]. Azeez and colleagues [73] reported moderate antibacterial activity against *Salmonella typhi* when ethanol leaf extracts, and crude hot and cold water were investigated *in-vivo*. Antibacterial activity was investigated in the aqueous leaf extract of P. nigrescens using the agar well diffusion method to detect the sensitivity of the extract against *Salmonella typhi*, *E. coli*, *Streptococcus mutans*, *Pseudomonas aeruginosa*, and *Staphylococcus aureus* at different concentrations of 50, 100, 200, and 300 mg/mL. The result showed that the ethanol leaf extract of *P. nigrescens* showed significant inhibition at a concentration of 300 mg/mL and lower inhibition at 50, 100, and 200 mg/mL against the organisms. This revealed that the ethanol leaf extract of *P. nigrescens* has the ability to kill microorganisms [74]. An ethanol leaf extract of *P. nigrescens* has been reported for antityphoid activity in Swiss albino mice. When mice infected with a standard inoculum of *Salmonella typhi* were treated with the ethanol extract at doses of 200 mg/mL, no signs of infection were observed after 7 days. This could be attributed to the antibacterial activity of the ethanol leaf extract of *P. nigrescens*. Meanwhile, signs of infection, including diarrhea, weakness, unformed stool, furring hair, and loss of weight, were observed with control mice inoculated with the standard inoculum of *Salmonella typhi* without the ethanol leaf extract of *P. nigrescens* after 7 days. There was a significant reduction in packed cell volume, red blood count, hemoglobin concentration, and mean corpuscular hemoglobin. Also, urobilinogen and bilirubin were present in the urine of the mice inoculated with the standard inoculum of *Salmonella typhi* without treatment. The urinalysis parameters were normal in the mice treated with the ethanol leaf extract of *P. nigrescens*. This could be attributed to the fact that the extract may have killed pathogens [6]. More studies are needed to confirm the extract with the highest potential of inhibition either in a laboratory and/or isolated strains of microorganisms that normally cause most infectious diseases in humans. There is a need for harmonization of *in-vitro* and *in-vivo* to justify the use of the plant in the treatment of infection. However, these studies conducted may be considered as preliminary scientific justification of the use of the plant in the treatment of infections such as measles, salmonella, whopping cough, gonorrhea, other sexually transmitted diseases, and noninfection related diseases such as diarrhoea, nausea, and vomiting.

### 3.6. Other Activities of *P. nigrescens*

In an *in-vitro* study, hydro-methanolic leaf extract of *P. nigrescens* was reported to exert a stimulating and spasmogenic effect on the myometrium of pregnant rats. This may be due to a rise in the magnitude of spontaneous isometric contractions and a minor increase in muscular contracture. The effect on myometrium exerted by the extract could be compared to that induced by sulprostone in pregnant rats. The result showed that the extract may possess an oxytocinlike property. An extracellular influx of calcium may be responsible for the increase in the extreme isometric contraction magnitude [75], which could justify the traditional use of the extract in childbirth. Oyelowo and colleagues [76] have also reported that the aqueous root extract of *P. nigrescens* in a male reproductive study enhanced male sexual reproductive behavior in Wistar rats. The extracts were administered at doses of 50, 100, and 150 mg/kg body weight. The frequency of mount, intromission, and ejaculation increased. The serum testosterone content improved in animals from day 1 to 5. The improvement in the sexual behavior could be attributed to the presence of phytochemicals such as flavanoids, alkaloids, and saponins, which have the ability to enhance androgen and antioxidant properties. In another study, aqueous leaf extract of *P. nigrescens* has also been reported to improve both physical and biochemical indices of male sexual activity, such as ejaculation, intromission, frequencies of the mount, latencies of the mount, and postejaculatory interval. The extract has the potential to restore changes in reproductive hormones and enzymes such as follicle stimulating hormones, testosterone, nitric oxide, and the activity of phosphodiesterase in paroxetine-induced sexual dysfunction in rats [21]. The administration of the aqueous leaf extract of *P. nigrescens* at a dose of 80 mg/kg body weight for 7 days significantly increased intromission, frequencies of mount, ejaculation, percentage of mounts, intromission ratio, copulatory efficiency, serum testosterone, follicle stimulating hormones, testosterone, and nitric oxide. Intromission, latencies of mount, intromission, postejaculatory interval, and the activity of phosphodiesterase V were significantly decreased and or shortened. Similar results were obtained in PowMaxM (reference drug)-treated rats. However, paroxetine treatment significantly reversed the results obtained from the aqueous leaf extract of *P. nigrescen* treatment when compared with increased and/or prolonged physical and biochemical indices of male sexual activity [21].

A methanol stem extract of *P. nigrescens* was evaluated in acute mouse models of cognition for memory-enhancing potential. The extracts were administered at doses of 250, 500, and 1000 mg/kg for assessment of learning and memory using the elevated plus maze, Barnes maze, and novel object recognition tests. The finding showed that the extract reduced the transferred latencies in the evaluated plus maze on days 1 and 2. Meanwhile, the escape latencies and escape errors in the Barnes maze were also reduced at doses of 250 and 1000 mg/kg, respectively. In addition, an increase in the target quadrant was observed at doses of 250 and 500 mg/kg. The discrimination index in novel object recognition tests was also increased with a reduced diazepam-induced transferred latencies on day 1. In the open field test, the extract in all doses did not cause significant changes in the number of rearing and square cross. The methanol stem extract could be a potential candidate for further studies in the treatment of memory loss and amnesia [77]. In another study, Aqueous leaf extract of *P. nigrescens* was given to female Wistar rats at three different doses (50, 100, and 200 mg/kg body weight) in a study aimed at investigating the usefulness of the plant in the treatment of polycystic ovarian syndrome (PCOS) induced by mifepristone. The result indicated that the aqueous leaf extract of *P. nigrescens* caused an increase in biochemical factors such as albumin, total protein, and liver aspartate aminotransferase activity. Creatinine, globulin, aminotransferase activities, serum alkaline phosphatase, total bilirubin, urea, and alanine in all the doses administered were reduced. The extract at a dose of 100 mg/kg body weight reduced the effect of mifepristone on the liver and kidney of the Wistar rats. No histological changes occurred in the organs such as the uterus, liver, and kidney of the mifepristonized rats treated with the extract [78].

*P. nigrescens* methanolic leaf extract and squalene isolates were investigated for gonadal function using arsenic trioxide-treated (As2O3) (arsenic-induced) reproductive toxicity in male Wistar rats for 54 days. Arsenic (metalloid) pollutes drinking water, causing male reproductive dysfunction by inducing oxidative stress. The result showed an increased in sperm motility when the rats were treated with arsenic plus 250 mg/kg methanol leaf extract of *P. nigrescens*, (91.2 ± 3.3%), arsenic plus 500 mg/kg methanol leaf extract of *P. nigrescens* (86.3 ± 2.8%), and arsenic plus 100 mg/kg squalene when compared to the control (distilled water 3 mg/kg) and 3 mg/kg of arsenic alone (70.94.5%). This could be attributed to the fact that *P. nigrescens* methanol leaf extract protects spermatozoa from the toxicity of arsenic trioxide to a certain degree. Moreso, normal sperm morphology was restored, which could be attributed to the spermatogenic protective ability of the methanol leaf extract when compared to the decrease in the percentage of normal morphology of spermatozoa in rats treated with arsenic (control untreated). Testicular histology and epididymal histology were improved when the methanol leaf extract of *P. nigrescens* and *squalene* were administered. Arsenic plus 250 mg/kg (1.00.1 nmol/L) and arsenic plus 500 mg/kg (1.00.1 nmol/L) increased testosterone concentrations when compared to arsenic alone (0.50.1 nmol/L). The finding showed that methanol leaf extract of *P. nigrescens* and *squalene* both ameliorated arsenic trioxide-induced reproductive toxicity in male Wistar rats [79].

In a study by Adesola and colleagues [80], the essential oil acquired by hydro-distillation of the leaf of *P. nigrescens* was diluted with dimethyl sulfoxide and evaluated for insecticidal potential using a conventional procedure. The result showed that the extract at a dose of 150 mg/mL concentration gave better fumigant toxicity against *Sitophilus zeamais* (maize weevil) with a 100% mortality rate after 72 hours. In addition, the concentrations such as 100, 200, and 250 mg/mL of the essential oil of *P. nigrescens* after 72 hours showed substantial toxicity against *S. zeamais* with an 80% mortality rate. The lethal concentrations (LC50) were 46.14 mg/mL of air compared to the control, permethrin, and allethrine (LC50 of 11.10 mg/mL and 7.40 mg/mL of air, respectively). *P. nigrescens* could be considered as a possible natural plant for the control of insect pests. The insecticidal activity observed in the essential oil of the plant may be attributed to the presence of neral and gerania, a compound isolated from the essential oil of *P. nigrescens* that has been reported to exhibit potential insecticidal activity [81, 82].

An herbal tea produced from *P. nigrescens* and *Cymbopogon citratus* (lemongrass) has been evaluated in Wistar rats at five different concentrations (100 : 0%, 87.5 : 12.5%, 75 : 25%, 62.5 : 37.5%, and 50 : 50%) for their effect on the health of the Wistar rats. The result showed that all the concentrations of the herbal tea significantly decreased blood urea nitrogen, triglycerides, low-density lipoprotein, alanine aspartate, body weight, alanine transaminase, and glucose. Also, no toxicity was observed when the kidney and liver were examined. There were significant increases in red blood cells and hemoglobin. The health benefits could be improved when *Parquetina*-lemongrass tea is taken because of its antidiabetic, erythropoietic, and anticholesteremic potential [83]. Hydroethanolic leaf extract of *P. nigrescens* has been investigated for testicular injury in male Wistar rats using a D-galactose-induced model. The extract was administered at a dose of 250 and 500 mg/kg body weight. The result showed improvement in relative testicular weight, level of testosterone, follicle stimulating hormone, and luteinizing hormone. The extract reduced the levels of malondialdehyde, advanced glycation end products, 8 hydroxy-deoxyguanosine (oxidative stress biomarkers), and inflammatory responses to normal. Also, the extract repaired the activity of superoxide dismutase, catalase, and glutathione peroxidase, lessened testicular DNA fragmentation, and down-regulated caspase 3-activities. Hydroethanolic leaf extract administered at 500 mg/kg body weight showed a better effect against D-galactose-induced testicular injury via anti-inflammatory, antiapoptotic, and antioxidative mechanisms. [37]. The aqueous and ethanolic root bark of *Parquetina nigrescens* have been investigated for the treatment of hypertension and edema for their diuretic properties in Wistar rats. The result showed that the aqueous extract administered at a dose of 15 mg/kg and the ethanol extract administered at a dose of 25 mg/kg caused an increase in urine excretion and an increase in urine volume when compared to that induced by furosemide (FURO 5 mg/kg). The urinary excretion concentration of 42.73 ± 0.26% (aqueous root bark extract), 45.39 ± 0.190% (ethanol root bark extract), and 73.6 ± 0.24% (standard drug-FURO) against saline solution-sodium chloride was 29.73 ± 0.24%. The increase in the urine volume excreted could ascetically justify the use of the plant in the treatment of hypertension and edema. Further studies are needed to clarify the mechanism of the diuretic activity of the plant [84].

## 4. Discussion

In this review, the parts of the plant mostly used for the pharmacological activities were the leaves (68%), followed by the stem bark and roots (11%), the whole plant and aerial parts (4%), and the fruit bark (2%). Several active compounds were isolated from the various parts of the plant. In summary, the literature search on the *in-vitro* and *in-vivo* work done on *P. nigrescens* revealed twenty-three (23) different pharmacological properties with respect to their possible experimental models, haematological and biochemical markers. The active dose (mg/kg) reported varied due to the variation in the dose worked on by the researchers and the experimental animal models used. A summary of the pharmacological activities, plant parts used, type of extract used, dose range and reported active dose, experimental animal models/haematological and biochemical markers used, and duration of the research has been presented in Table 2:

Medicinal plants remain one of the main sources of present-day medicine. The belief that herbal products prepared from medicinal plants are safe because they are from natural sources is not always accurate. Awareness of medicinal plant pharmacognosy has improved due to the use of herbal therapy as an alternative to orthodox medicine in recent times [23]. The improvement in research into the pharmacological activities of medicinal plants gives proper scientific evidence to establish the efficacy, safety, and toxicological profile of commonly used medicinal plants. The *in-vivo* and *in-vitro* studies of medicinal plants, especially the toxicity, help to provide much needed data on the safety of medicinal plants when the plants are found to have the adequate potential for development into therapeutic drugs and chemicals. This review has also presented some toxicity studies of *P. nigrescens,* as reported by some researchers in Table 3: Reported cases of *P. nigrescens* toxicological assessment in mice. Checks on the safety and toxicological profiles of medicinal plants are necessary to promote their safe use.

## 5. Future Prospects

- The lethal concentration 50 (LC_50_) or the mean lethal dose 50 (LD_50_) which is the amount of a substance required to kill 50% of test animals during a predetermined observation period, should be well documented in each experiment.
- There were variations in the doses and duration selected by the researchers. Confirmation of the actual dose per each model should be clear in the future.
- The number of test animals used must be literature-specific so as to rule out errors.
- The active compounds isolated from *nigrescens* should further be evaluated for individual pharmacological activities. Isolation of more compounds from the plant should also continue to help in the development of novel drugs.
- The parts of the plant, such as roots, fruits, latex, leaf sap, and flowers, should be investigated scientifically in order to confirm their traditional uses since most of the research focused on the leaves and stems. In addition, other traditional uses, such as use in the treatment of epilepsy, piles, lower back pain, jaundice, conjunctivitis, and as a cardiac tonic, which have not been scientifically studied, can be studied to justify the use of the plant for the respective disease conditions.

## 6. Conclusion and Recommendation

In the current review, it was observed that *P. nigrescens* is rich in phytochemical constituents and possesses pharmacological properties. Hence, it might be considered as a remedy for different diseases such as anemia, diabetes, ulcers, inflammation, asthma, and typhoid. The study recommends that further investigation should focus on the identification, separation, purification, and quantification of the most bioactive compounds present in the plant to ascertain its usefulness in the pharmaceutical industry. In addition, more studies are needed on practical doses in animals and humans. Clinical observation and trials may help to determine effective dosage regimes of the plant.

## Figures and Tables

**Table 1:** Summary of claim traditional uses of part of *P. nigrescens* and types of preparation.

Plant part used	Type of preparation	Traditional uses
Leaves	Milled and mixed with honey	Fatigue [21]
	Decoction	Diabetes, menstrual disorders, stomach ulcer, diarrhea, insanity, gastric ulcer, asthma, sickling cell anemia, salmonella, and whopping cough [6, 7, 9, 23–25]
	Pounded and macerated fresh leaves	Headaches, fatigue [26], head lice, and skin diseases. [3]
	Infusion	Pile and lower back pain [9] wound healing [5]
	Powder	Supporting immune system

Roots	Decoction	Menstrual pain, gonorrhea, and nicotine poisoning [5]
	Infusion	Dysentery, measles, intestinal worms, and snake bite [5]
	Crush/milled	Venereal disease [5]
	Crush/milled in olive oil or shea butter (*Vitellaria paradoxa*)	Snake and insect repellent [5, 9]
	Paste	Convulsion, vomiting, nausea, and epilepsies [5]
	Pulverized roots	Aphrodisiac when applied on the body [28]
Stem bark	Decoction	Cardiac tonic [5] and aphrodisiac [29]
	Pulverized stem bark	Arthritis and rheumatism [28]

Flowers	Greenish outside, deep red inside	Beautification and aesthetic purposes [33]
Latex and leaf sap	Milky fluid	Wound, abscesses., burns treatments, and tumors [32] venereal diseases
Leaves sap	Milky fluid	Jaundice and conjunctivitis [31]
Latex	White, hardening to black	Arrow poison for hunting bush meat and production of black rubber [33]

source: Summary of claim traditional uses of parts of the plant was generated by Authors'.

**Table 2:** Pharmacological activity (*in-vitro* and *in-vivo*) of *P. nigrescens*.

Pharmacological activity	Plant part	Type of extract	Isolated compound tested	Dose range (mg/kg)	Reported active dose (mg/kg)	Experimental models/haematological and biochemical markers	Duration of the experiment	References
Antianemic	Leaves	Aqueous		2000–2500	2500	Phenylhydrazine-induced	7 days	[41]
	Leaves	Aqueous		1000	1000	Alloxan monohydrate-induced	14 weeks	[25]
Antidiabetic	Whole plant	Aqueous		200–800	400, 800	Streptozotocin–nicotinamide-induced	14 weeks	[48]
	Leaves	Aqueous		0–1000	1000	Alloxan-induced (5%w/v)	4 weeks	[25]
		Methanol		100–200	200	Alloxan-induced (120 mg/kg)	4 weeks	[42]
		Methanol		100–200	100	Alloxan-induced (120 mg/kg)	4 weeks	[33, 42]
		Methanol	Phytol	250	250	Alloxan-induced (120 mg/kg)	4 weeks	[33]
		Methanol	Squalene	3	3	Alloxan-induced (120 mg/kg)	4 weeks	[33]
	Root bark	Ethyl acetate fraction	Convallatoxin	20	20	Streptozotocin-induced diabetic	10 days	[50]
Antihyperlipidemic	Whole plant	Aqueous		200–800	200, 400	Streptozotocin–nicotinamide-induced	14 days	[48]
	Root	Aqueous		50–150	50	Automated analyzer (roche, HITACHI).	21 days	[26]
Antioxidant	Leaves	Aqueous		50	50	Fe2+/ascorbate-induced	9 days	[3]
	Stem bark	Methanol		50	50	Fe2+/ascorbate-induced	9 days	[3]
		Methanol (ethyl acetate fraction)	Flavonoid	50	50	Fe2+/ascorbate-induced	9 days	[3]
	Roots	Aqueous		50–150	100	RANSOD kit (randox, crumlin, England)	21 days	[26]
	Leaves and stem	Essential oil		0.25–1.0	1.0	2, 2-diphenylpicryl hydrazine (DPPH) radical scavenging assay	48 hours	[53]
	Leaves	Methanol		500–1000	500	Ischemia-reperfusion induced	7 days	[52]
	Leaves	Ethanol		100–200	100	Isoproterenol-induced (85 mg/kg)	6 weeks	[54]
	Aerial parts	Polyphenol-rich fraction		100–200	200	Dichlorvos-induced cardio- and renal toxicity	14 days	[28]
Antiulcerogenic	Leaves	Hexane		500–1000	500	Ethanol-induced gastric ulceration	14 days	[55]
		Chloroform		500–1000	1000	Ethanol-induced gastric ulceration	14 days	[55]
		Aqueous		100–500	100	Ethanol-induced gastric ulceration	14 days	[5]
Antimicrobial	Leaves	Aqueous		6–18	3 to >18	Antibiotic susceptibility test; agar diffusion method, spread plate method	28–48 hours	[5]
		Ethanol		6–18	15 to >18	Antibiotic susceptibility test; agar diffusion method, spread plate method	28–48 hours	[5]
		Aqueous		50–200	50	Antibiotic susceptibility test; spread plate method	21 days	[71]
		Ethanol		50–200	200	Antibiotic susceptibility test; spread plate method	21 days	[71]
	Leaves	Aqueous		50–300	300, 200	Antibiotic susceptibility; agar well diffusion method	28–48 hours	[74]
		Ethanol		200	200	Standard inoculum of salmonella typhi	7 days	[6]
Antisickling	Leaves and stem	Aqueous-methanol		0–5	5	Sodium metabisulphite -induced	14 days	[23]
	Leaves	Hydroethanolic		0.05–10	10	Sodium metabisulfite- induced	14 days	[57]
Antimalarial	Leaves	Aqueous		150	150	Standard inocula (5×106) of chloroquine sensitive plasmodium berghei (NK 65)	18 days	[59]
Antidiarrheal	Roots	Methanol		25–100	100	Castor oil-induced diarrhea	28 days	[34]
	Leaves	Methanol		100–1000		Castor oil-induced diarrhea *E. coli* induced diarrhea	28 days	[40]
	Leaves	Aqueous–methanol		5–200	200	Castor oil-induced diarrhea	4 weeks	[63]
Anti-inflammatory	Leaves	Aqueous		50–200	200	Cotton pellet granuloma- induced carrageenan oedema -induced formaldehyde- induced	4 weeks	[64]
Analgesic activity	Leaves	Aqueous		50–200	200	Formalin paw licking- induced hot plate latency- induced	4 weeks	[64]
Antipyretic	Leaves	Aqueous		50–200	200	brewer's yeast- induced	4 weeks	[64]
Antinociceptive	Fruit bark	Methanol		50–200	200	Formalin and acetic acid-induced	4 weeks	[65]
asthmatic	Leaves	Aqueous		400–800	400	Antigen-induced broncho spasms	14 days	[62]
Toxicity	Leaves	Aqueous		2000–8000	400	Brine shrimp lethality bioassay and Lorke's method	29 days	[43]
	Leaves	Aqueous		400–1600	800	Haematological and biochemical analysis, randox test kit, cynomethaemoglobin method	6 weeks	[27]
	Leaves	Methanol		500–5000	All	Alloxan monohydrate-induced	28 days	[42]
	Leaves and aerial part	Methanol		100–1000	1000	Haematological and biochemical analysis, HPLC profile	35 days	[68]
Cytotoxicity	Leaves	Methanol		0.1–100	100	HaCaT keratinocytes	35 days	[68]
	Leaves	Ethanol		0.1–100	8.33	(3-(4, 5- dimethylthiazol-2-yl)-2,5-diphenyltetrazolium bromide (MTT) assay, methylene blue and trypan blue exclusion assay	72 hours	[69]
	Leaves	Essential oil		10–1000	100, 1000	Brine shrimp lethality assay	48 hours	[53]
	Stem	Essential oil		10–1000	1000	Brine shrimp lethality assay	48 hours	[53]
	Leaves	Aqueous		1–50	10, 20	Allium cepa assay	14 days	[46]
Antineurotoxicity	Leaves	Hydroethanolic		250–500	500	D-galactose-induced	6 Weeks	[70]
Aphrodisiac	Root	Aqueous		50–150	100	Heamatological and biochemical analysis	3 weeks	[76]
	Leaves	Aqueous		20–80	80	Paroxetine hydrochloride- induced	7 days	[21]
Antiamnesiac	Stem	Methanol		250–1000	250, 500	Elevated plus maze, barnes maze and novel object recognition test, open field test, and diazepam induced amnesia	14 days	[77]
Polycystic ovarian syndrome	Leaves	Aqueous		50–200	100	Mifepristone-induced (7.14 mg/kg)	30 days	[78]
Anti-insecticidal	Leaves	Hydro-distillation essential oil		50–250	150, 200, 250	Maize weevil (sitophilus zeamais)	72 hours	[82]
testicular injury	Leaves	Hydroethanolic		250–500	500	D-galactose-induced	6 weeks	[37]

Source: Pharmacological activity (*in-vitro* and *in-vivo*) of *P. nigrescens* was generated by the Authors'.

**Table 3:** Reported cases of *P. nigrescens* toxicological assessment in mice.

Plant parts	Type of extraction	Experimental model/method	No. and names of test animals	Actual dosages administered	Dosages reported with toxic effects	Mortality	Duration of experiment	LC_50_(mg/kg)/(*μ*g/mL)	Organs/body fluids affected	References
Whole	Reflux condensation		6 sprague-Dawley rats	400, 800, 1600 (mg/kg)	1600 mg/kg·p.o^a^^*∗*^	None	6 weeks		Decreases WBC	[27]
Leaves	Aqueous extract	Brine shrimp lethality bioassay and toxicities (Lorke's method)	27 swiss albino mice (male)	3000, 6000, 12000 (mg/kg)	12000 mg/kgbw^b^^*∗∗*^	Yes (2/3) mice	28 days	32.34 *μ*g/mL	Mild kidney interstitial fibrosis, mild glomerular hypercellularity, and mild liver microhemorrhages.	[43]
Leaf, aerial	Methanol extract	HaCaT keratinocytes	50 Wistar rats (male)	100, 300, 1000 (mg/kg)	100, 300 (mg/kg)	None	35 days		Renal hemorrhage and hepatic inflammation	[68]
Leaf, aerial	Methanol extract	HaCaT keratinocytes	50 Wistar rats (male)	100, 300, 1000 (mg/kg)	100,300, 1000 (mg/kg)	None	35 days		Decreased WBC	[68]
Root			40 Wistar rats (male)	50, 100, 150 (mg/kg)	50,100 (mg/kg)		21 days		Decreased WBC	[26]
Root		Automated haematological analyzer systemex KX-21 (Japan)	40 wistar rats (male)	50, 100, 150 (mg/kg)	50 mg/kg		21 days		Decreased Eosinophil count	[26]

a^*∗*^ = acute; b^*∗∗*^ = sub-acute. Source: Reported cases of *P. nigrescens* toxicological assessment in mice was generated by the Authors.

## Data Availability

The data used to support the findings of this study are included within the article.
